# Motor Vehicle Traffic-Related Pedestrian Deaths — United States, 2001–2010

**Published:** 2013-04-19

**Authors:** Rebecca B. Naumann, Laurie F. Beck

**Affiliations:** Div of Unintentional Injury Prevention, National Center for Injury Prevention and Control, CDC

Motor vehicle traffic crashes are the leading cause of unintentional injury-related death in the United States, resulting in 33,687 deaths in 2010 (1). Pedestrian travel makes up 10.5% of all trips (i.e., any travel from one address to another) taken in the United States, and pedestrians represent 13% of all motor vehicle traffic-related deaths (1,2). To determine traffic-related pedestrian death rates by sex, age group, race/ethnicity, and urbanization level, CDC analyzed 2001–2010 data from the National Vital Statistics System (NVSS). The results of that analysis indicated that the overall, annualized, age-adjusted traffic-related pedestrian death rate was 1.58 deaths per 100,000 population. Persons aged ≥75 years and those categorized as American Indian/Alaska Native (AI/AN) had the highest death rates, and age group differences varied by race/ethnicity. The results suggest that the overall pedestrian death rate could increase with the aging and growing racial/ethnic diversity of the U.S. population. The U.S. Census Bureau projects that the number of persons aged ≥75 years will more than double, from approximately 18 million in 2011 (6% of the U.S. population) to 44 million in 2040 (12% of the population); minority racial/ethnic populations are projected to increase from 116 million in 2010 (37% of the population) to 186 million in 2040 (49% of the population).* Strategies to prevent pedestrian deaths should include consideration of the needs of older adults and cultural differences among racial/ethnic populations.

NVSS data were accessed through CDC WONDER, which provides customized reports of mortality data, and information on other health outcomes and risk factors (e.g., birth data and sexually transmitted disease morbidity).† NVSS collects death certificate data from vital statistics offices in all 50 states and the District of Columbia.§ Motor vehicle traffic-related pedestrian deaths were defined as any deaths for which the underlying cause recorded on death certificates was one of the following *International Classification of Diseases, 10th Revision* codes: V02–V04 (.1,.9) or V09.2.¶ Pedestrian deaths and annualized death rates per 100,000 population for the years 2001–2010 were examined by sex, age group, race/ethnicity, and urbanization level. Annualized death rates for sex, race/ethnicity, and urbanization level were age-adjusted to the 2000 standard U.S. population. Traffic-related pedestrian death counts less than 20 (and the associated rates) were not reported for racial/ethnic populations because of concerns regarding statistical reliability and data confidentiality. However, such counts were included in the statistics for all pedestrians combined.

Race/ethnicity was coded into five mutually exclusive categories: white, black, AI/AN, Asian/Pacific Islander (A/PI), and Hispanic. All persons categorized in the first four groups were non-Hispanic. Persons categorized as Hispanic might be of any race. Urbanization was categorized into six levels of area: large central metro, large fringe metro, medium metro, small metro, micropolitan (nonmetro), and noncore (nonmetro).**

During 2001–2010, a total of 47,392 pedestrians (32,873 males and 14,519 females) died from traffic crashes (Table 1). The overall, annualized age-adjusted traffic-related pedestrian death rate was 1.58 deaths per 100,000 population. The age-adjusted death rate for males (2.29) was 2.5 times the rate for females (0.92). Pedestrian death rates increased with age. For males, death rates were highest among those aged ≥85 years (6.35), followed by those aged 75–84 years (4.53); rates were lowest among those aged 0–14 years (0.83), followed by those aged 15–24 years (1.98). For females, death rates were highest among those aged 75–84 years (2.43), followed by those aged ≥85 years (2.16); rates were lowest among those aged 0–14 years (0.43), followed by those aged 25–34 years (0.72) and 15–24 years (0.78).

AI/ANs, among both males (7.73) and females (2.22), had the highest annualized, age-adjusted traffic-related pedestrian death rates of all races/ethnicities (Table 1). For males, Hispanics and blacks had the next highest death rates (3.93 and 3.73, respectively), followed by A/PIs (1.96). For females, A/PIs had the second highest death rate (1.46), followed by blacks (1.31) and Hispanics (1.27). Among both males (1.78) and females (0.79), whites had the lowest pedestrian death rates. By urbanization level, among both males (2.90) and females (1.23), those living in large central metro areas had the highest pedestrian death rates.

For males in the 15–24, 25–34, 35–44, and 45–54 year age groups, racial/ethnic disparity patterns generally were similar (Figure). In each of these age groups, the highest death rates were among AI/ANs (range: 8.13–11.72), followed by blacks (2.29–5.97) and Hispanics (2.61–4.60). Whites (range: 1.66–2.28) and A/PIs (0.70–1.36) had the lowest death rates. For males aged 75–84 and ≥85 years, Hispanic (11.05 and 14.70, respectively) and A/PI (12.30 and 20.53, respectively) death rates were statistically greater than the rates for whites (3.61 and 5.41, respectively) and blacks (6.78 and 6.95, respectively).

Among females, AI/ANs also had the highest death rates for each of the age groups 15–24, 25–34, 35–44, and 45–54 years (Figure). Across those age groups, the death rate for AI/ANs ranged from 2.29 to 4.17, and was followed by the rate for blacks (range: 0.96–1.88). Hispanics (range: 0.62–1.15), whites (0.68–0.86), and A/PIs (0.55–0.97) had similar death rates. For females aged 75–84 and ≥85 years, Hispanic (5.33 and 4.03, respectively) and A/PI (8.82 and 6.87, respectively) death rates were statistically greater than the rates for whites (2.06 and 2.02, respectively) and blacks (1.94 and 1.36, respectively).

Pedestrian death rates generally increased with age across all six urbanization levels (Table 2). In large central metro areas, those aged 35–44 years (2.08), 45–54 years (2.60), 55–64 years (2.60), 65–74 years (3.36), 75–84 years (5.19), and ≥85 years (5.24) had statistically higher death rates than those in the same age groups at other urbanization levels. By race/ethnicity, in large central metro areas, death rates for whites (1.57) and Hispanics (2.74) were statistically greater than in nonmetro areas (whites, micropolitan: 1.18 and whites, noncore: 1.13; Hispanics, micropolitan: 2.26 and Hispanics, noncore: 1.89). However, the rates for other races/ethnicities, notably AI/ANs, did not follow this pattern. For example, the pedestrian death rate for AI/ANs living in noncore (nonmetro) areas (7.04) was approximately twice that for AI/ANs living in large central metro areas (3.58).

## Editorial Note

This report examines annualized motor vehicle traffic-related pedestrian death rates by key sociodemographic variables. The results indicated that, among racial/ethnic populations, AI/ANs had the highest traffic-related pedestrian death rates, and by age group, persons aged ≥75 years had the highest rates. Age-related patterns in death rates varied by race/ethnicity.

These results support those from previous research showing that males consistently have higher traffic-related pedestrian death rates than females (3). Recent research has shown that, on average, males and females walk similar distances, and although males have a slightly higher risk for being involved in a collision as a pedestrian, the observed differences have been found largely driven by a higher case-fatality rate among males than females (4). Some researchers have speculated that males exhibit riskier pedestrian behaviors or walk in more dangerous settings, but little research has explored the differences by sex in pedestrian death rates.

Among both males and females, pedestrian death rates generally increased with age. The highest death rates for both sexes were observed among those aged 75–84 and ≥85 years. Studies of travel behavior have found that older adults take fewer walking trips and walk, on average, fewer miles per year than younger persons (2); however, when struck, older adult pedestrians are more likely than younger adults to die from their injuries (5). Higher prevalence of chronic disease, disability, and frailty among older adults might contribute to these higher case-fatality rates. In addition, age-related declines in cognitive functioning, vision, and physical functioning might place older adult pedestrians at greater risk for being struck by a vehicle. For example, older adults take longer than younger adults to cross roadways (6).

Previous research also has found that certain racial/ethnic populations are disproportionally affected by pedestrian crashes (7). The current study supports these differences, including the fact that among all ages combined, AI/ANs had higher death rates than persons in other racial/ethnic populations. The study further found that racial/ethnic patterns in pedestrian death rates differed across age groups. Research findings are mixed regarding why certain racial/ethnic populations have higher death rates. A report on 2006 U.S. traffic fatality data showed that higher percentages of AI/AN pedestrians and pedalcyclists who died in motor vehicle traffic crashes had some level of alcohol or a blood alcohol concentration of ≥0.8 g/dL, compared with pedestrians and pedalcyclists of other races/ethnicities (7). Other research has shown that increased risks remain for certain minority populations, even after controlling for lower socioeconomic status, increased exposure to traffic, and increased use of alcohol (8). Additional research is needed to understand the factors that place certain racial/ethnic populations at increased risk for pedestrian death, and the patterns in racial/ethnic differences by age group.

What is already known on this topic?

Motor vehicle traffic crashes are the leading cause of unintentional injury-related death in the United States. Pedestrians are particularly vulnerable road users and disproportionally represented in motor vehicle traffic deaths.

What is added by this report?

Adults aged ≥75 years and American Indians/Alaska Natives had the highest traffic-related pedestrian death rates in the United States during 2001–2010. Age-related patterns in traffic-related pedestrian death rates differed by race/ethnicity.

What are the implications for public health practice?

The overall pedestrian death rate could increase given the aging and growing racial/ethnic diversity of the U.S. population. Strategies to prevent traffic-related pedestrian deaths should consider the needs of older adults as well as persons of different races and ethnicities.

Approximately three fourths of all pedestrian deaths in 2010 occurred in urban areas (3). Higher pedestrian death rates in urban areas are, at least in part, a result of more concentrated vehicle and pedestrian activity in these areas. The current study found that for many age groups and racial/ethnic populations, patterns in pedestrian death rates by level of urbanization were similar to those for overall pedestrian death rates and further found that the differences in pedestrian deaths when comparing large central metro and noncore (nonmetro) areas were most pronounced among adults ≥65 years. In contrast, death rates among AI/ANs were higher in nonmetro areas than in large central metro areas, which is consistent with previous research (9).

The findings in this report are subject to at least five limitations. First, vehicle, driver, and roadway characteristics (e.g., vehicle speed, driver alcohol use, and traffic density) that are known risk factors for crash-related deaths were not available from NVSS. Second, the small numbers of pedestrian deaths among certain groups (i.e., AI/AN females aged ≥65 years, AI/AN males aged ≥75 years) prevented estimation of death rates among these groups. Third, the urbanization level variable is defined as the county of the person’s legal residence, not the county where the crash occurred. Fourth, for some motor vehicle traffic-related deaths, the road user type (e.g., occupant, pedestrian, pedalcyclist, or motorcyclist) was unknown; therefore, pedestrian death rates might be underestimated. Finally, because NVSS data are extracted from death certificates, some racial misclassification is likely. This can result in underestimated death rates for some minority populations, particularly AI/ANs (10).

Approximately 4,000 pedestrians die from crash-related injuries each year in the United States, and certain populations are disproportionately affected (1). Addressing the risks that pedestrians of different ages, sexes, and races/ethnicities face in various settings requires a multifaceted approach. *Pedestrian Safety: a Road Safety Manual for Decision-Makers and Practitioners* will be released by the World Health Organization to coincide with Global Road Safety Week (May 6–12, 2013), which this year focuses on pedestrian safety. The manual will include effective strategies for reducing pedestrian deaths such as roadway engineering improvements (e.g., installing and/or upgrading crosswalks, sidewalks, and raised medians); slowing vehicle speeds by implementing traffic calming measures (e.g., speed humps); enforcing speeding, distracted driving, and pedestrian-right-of-way laws; creating pedestrian safety zones and streets designated for walking; and improving mass transit route design and access. Many of these strategies can help prevent pedestrian deaths among high-risk older adults, but additional approaches that are specific to older adults also might be needed (e.g., longer pedestrian walk signals). Research on how to effectively tailor strategies (e.g., community outreach and media campaigns) to minority populations also is needed.

## Figures and Tables

**FIGURE:**
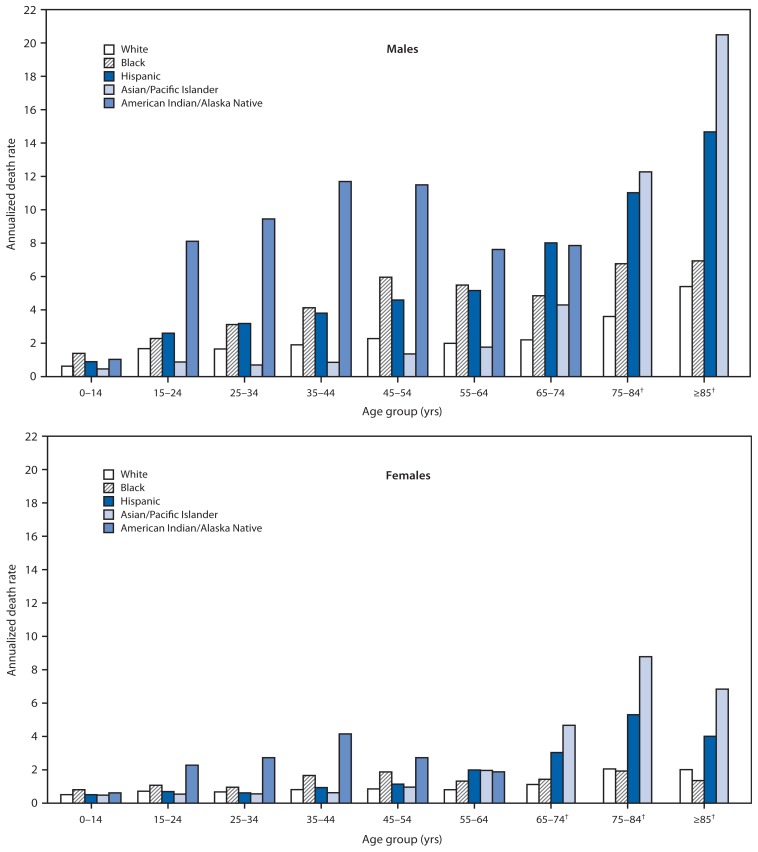
Annualized motor vehicle traffic-related pedestrian death rates^*^ for males and females, by age group and race/ethnicity — National Vital Statistics System, United States, 2001–2010 ^*^ Per 100,000 population. ^†^ Data not shown for American Indian/Alaska Natives in these age groups because of small numbers.

**TABLE 1:** Number of motor vehicle traffic-related pedestrian deaths and annualized death rates,* by sex and selected characteristics — National Vital Statistics System, United States, 2001–2010

	Males			Females		
		
Characteristic	No.	Annualized death rate	(95% CI)	No.	Annualized death rate	(95% CI)
**Overall**	**32,873**	**2.29**	**(2.26–2.31)**	**14,519**	**0.92**	**(0.90–0.93)**
**Age group (yrs)**						
0–14	2,496	0.83	(0.80–0.86)	1,353	0.43	(0.41–0.46)
15–24	4,308	1.98	(1.92–2.04)	1,616	0.78	(0.74–0.82)
25–34	4,349	2.18	(2.12–2.24)	1,428	0.72	(0.68–0.76)
35–44	5,399	2.51	(2.44–2.58)	2,122	0.98	(0.94–1.02)
45–54	6,278	3.00	(2.93–3.07)	2,250	1.04	(1.00–1.08)
55–64	3,958	2.64	(2.56–2.72)	1,641	1.02	(0.97–1.07)
65–74	2,658	2.96	(2.85–3.07)	1,507	1.43	(1.36–1.50)
75–84	2,399	4.53	(4.35–4.71)	1,866	2.43	(2.32–2.54)
≥85	953	6.35	(5.95–6.75)	719	2.16	(2.00–2.32)
**Race/Ethnicity**						
White	17,839	1.78	(1.76–1.81)	8,659	0.79	(0.78–0.81)
Black	6,063	3.73	(3.63–3.83)	2,484	1.31	(1.26–1.36)
Hispanic	6,809	3.93	(3.82–4.04)	2,120	1.27	(1.22–1.33)
A/PI	966	1.96	(1.83–2.10)	914	1.46	(1.36–1.56)
AI/AN	891	7.73	(7.20–8.26)	272	2.22	(1.95–2.49)
**Urbanization level of area**						
Large central metro	11,843	2.90	(2.85–2.96)	5,558	1.23	(1.19–1.26)
Large fringe metro	6,564	1.90	(1.85–1.94)	3,031	0.81	(0.78–0.84)
Medium metro	6,460	2.26	(2.21–2.32)	2,719	0.88	(0.84–0.91)
Small metro	2,796	2.02	(1.95–2.10)	1,190	0.82	(0.77–0.86)
Micropolitan (nonmetro)	3,138	2.08	(2.00–2.15)	1,272	0.82	(0.77–0.86)
Noncore (nonmetro)	2,072	2.16	(2.07–2.26)	749	0.75	(0.70–0.81)

**Abbreviations:** CI = confidence interval; A/PI = Asian/Pacific Islander; AI/AN = American Indian/Alaska Native.

* Per 100,000 population. Death rates overall and by race/ethnicity and urbanization level are age-adjusted.

**TABLE 2:** Number of motor vehicle traffic-related pedestrian deaths and annualized death rates,* by urbanization level of area, age group, and race/ethnicity — National Vital Statistics System, United States, 2001–2010

	Large central metro		Large fringe metro		Medium metro		Small metro		Micropolitan (nonmetro)		Noncore (nonmetro)	
						
Characteristic	No.	Annualized death rate (95% CI)	No.	Annualized death rate (95% CI)	No.	Annualized death rate (95% CI)	No.	Annualized death rate (95% CI)	No.	Annualized death rate (95% CI)	No.	Annualized death rate (95% CI)
**Overall**	**17,401**	**2.01 (1.98–2.04)**	**9,595**	**1.34 (1.31–1.37)**	**9,179**	**1.52 (1.49–1.55)**	**3,986**	**1.38 (1.34–1.43)**	**4,410**	**1.42 (1.38–1.46)**	**2,821**	**1.47 (1.41–1.52)**
**Age group (yrs)**												
0–14	1,296	0.72 (0.68–0.76)	724	0.50 (0.46–0.54)	810	0.65 (0.60–0.69)	366	0.64 (0.57–0.70)	405	0.67 (0.61–0.74)	248	0.69 (0.60–0.78)
15–24	1,821	1.44 (1.37–1.51)	1,292	1.38 (1.30–1.46)	1,155	1.32 (1.24–1.40)	561	1.22 (1.12–1.32)	631	1.42 (1.31–1.53)	464	1.86 (1.69–2.03)
25–34	2,044	1.51 (1.44–1.58)	1,197	1.30 (1.23–1.37)	1,104	1.44 (1.36–1.52)	499	1.39 (1.27–1.51)	552	1.54 (1.41–1.67)	381	1.78 (1.60–1.96)
35–44	2,722	2.08 (2.00–2.16)	1,476	1.31 (1.24–1.38)	1,496	1.79 (1.70–1.88)	629	1.64 (1.51–1.77)	737	1.80 (1.67–1.93)	461	1.78 (1.62–1.94)
45–54	3,123	2.60 (2.51–2.69)	1,726	1.59 (1.51–1.67)	1,715	2.03 (1.93–2.13)	772	1.94 (1.80–2.08)	752	1.71 (1.59–1.83)	440	1.52 (1.38–1.66)
55–64	2,176	2.60 (2.49–2.71)	1,177	1.56 (1.47–1.65)	1,036	1.66 (1.56–1.76)	446	1.48 (1.34–1.62)	470	1.34 (1.22–1.46)	294	1.22 (1.08–1.36)
65–74	1,702	3.36 (3.20–3.52)	819	1.85 (1.72–1.98)	769	1.94 (1.80–2.08)	303	1.55 (1.38–1.72)	355	1.49 (1.34–1.64)	217	1.27 (1.10–1.44)
75–84	1,794	5.19 (4.95–5.43)	841	2.87 (2.68–3.06)	778	2.91 (2.71–3.11)	286	2.23 (1.97–2.49)	353	2.29 (2.05–2.53)	213	1.97 (1.71–2.23)
≥85	681	5.24 (4.85–5.63)	331	3.01 (2.69–3.33)	295	3.01 (2.67–3.35)	119	2.52 (2.07–2.97)	145	2.54 (2.13–2.95)	101	2.46 (1.98–2.94)
**Race/Ethnicity**												
White	7,335	1.57 (1.53–1.61)	6,151	1.14 (1.11–1.17)	5,489	1.25 (1.21–1.28)	2,676	1.20 (1.15–1.24)	2,998	1.18 (1.13–1.22)	1,849	1.13 (1.08–1.18)
Black	3,892	2.57 (2.49–2.65)	1,548	2.01 (1.90–2.11)	1,460	2.38 (2.26–2.51)	579	2.19 (2.00–2.37)	652	2.60 (2.39–2.80)	416	2.62 (2.37–2.87)
Hispanic	4,715	2.74 (2.65–2.83)	1,430	2.27 (2.13–2.41)	1,703	2.64 (2.50–2.78)	470	2.10 (1.89–2.31)	432	2.26 (2.02–2.50)	179	1.89 (1.59–2.19)
A/PI	1,095	1.83 (1.72–1.94)	344	1.34 (1.18–1.49)	298	1.54 (1.36–1.71)	55	1.47 (1.04–2.01)	77	1.72 (1.36–2.16)	—†	—
AI/AN	142	3.58 (2.97–4.18)	71	2.55 (1.96–3.25)	180	4.18 (3.53–4.82)	186	6.24 (5.31–7.16)	226	5.09 (4.41–5.77)	358	7.04 (6.29–7.79)

**Abbreviations:** CI = confidence interval; A/PI = Asian/Pacific Islander; AI/AN = American Indian/Alaska Native.

* Per 100,000 population. Death rates overall and by race/ethnicity are age-adjusted.

† Data not shown because of small numbers.

## Footnotes

* Population estimates and projections available at http://www.census.gov.

† Available at http://wonder.cdc.gov.

§ Additional information available at http://www.cdc.gov/nchs/deaths.htm.

¶ Includes pedestrians injured in traffic collisions with cars, pick-up trucks, vans, two- or three-wheeled motor vehicles, heavy transport vehicles, buses, and other motor vehicles.

** Additional information available at http://wonder.cdc.gov/wonder/help/cmf/urbanization-methodology.html.

## References

[1] CDC (2013). Web-based Injury Statistics Query and Reporting System (WISQARS).

[2] Pucher J, Buehler R, Merom D, Bauman A (2011). Walking and cycling in the United States, 2001–2009: evidence from the National Household Travel Surveys. Am J Public Health.

[3] National Highway Traffic Safety Administration (2012). Traffic safety facts, 2010 data: pedestrians.

[4] Zhu M, Zhao S, Coben JH, Smith GS (2012). Why more male pedestrians die in vehicle-pedestrian collisions than female pedestrians: a decompositional analysis. Inj Prev.

[5] National Highway Traffic Safety Administration (2008). National pedestrian crash report.

[6] Avineri E, Shinar D, Susilo YO (2012). Pedestrians’ behaviour in cross walks: the effects of fear of falling and age. Accid Anal Prev.

[7] National Highway Traffic Safety Administration (2009). Traffic safety facts, 2006 data: race and ethnicity.

[8] Chen C, Lin H, Loo BP (2012). Exploring the impacts of safety culture on immigrants’ vulnerability in non-motorized crashes: a cross-sectional study. J Urban Health.

[9] LaValley J, Crandall CS, Banks L, Sklar DP, Boodlal L (2003). Rural and urban fatal pedestrian crashes among United States American Indians and Alaskan Natives. Annu Proc Assoc Adv Automot Med.

[10] Rosenberg HM, Maurer JD, Sorlie PD (1999). Quality of death rates by race and Hispanic origin: a summary of current research, 1999. Vital Health Stat 2.

